# Calcifications in multiple lymph node chains

**DOI:** 10.36416/1806-3756/e20240152

**Published:** 2024-07-29

**Authors:** Edson Marchiori, Bruno Hochhegger, Gláucia Zanetti

**Affiliations:** 1. Universidade Federal do Rio de Janeiro, Rio de Janeiro (RJ) Brasil.; 2. University of Florida, Gainesville (FL) USA.

**Figure 1:**
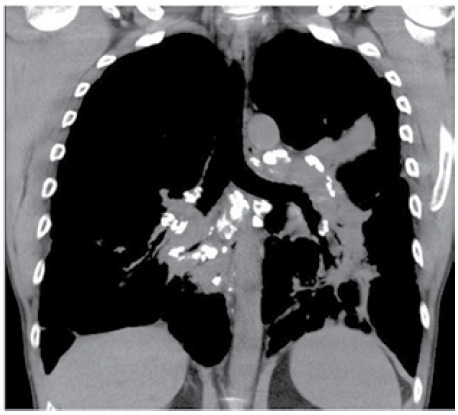
Coronal reconstruction of a chest CT scan with a mediastinum window setting showing calcifications affecting lymph nodes in several mediastinal and hilar chains. Also note evidence of fibrotic distortion in the perihilar regions.

A 46-year-old female patient with no smoking history, complaining of dry cough and tiredness, was admitted. Her laboratory test results were unremarkable. Chest CT showed reticular opacities with architectural distortion predominating in the middle thirds of the lungs, in addition to lymph node calcifications affecting multiple mediastinal and hilar chains (Figure 1).

Lymph node calcifications are most often sequelae of granulomatous infections, especially tuberculosis and histoplasmosis. Other less common causes are sarcoidosis, silicosis, amyloidosis, and calcifications secondary to lymphoma treatment (radiotherapy or chemotherapy).

However, the characteristics of lymph node calcifications in the patient were very specific. The calcifications involved multiple lymph node chains. When the presence of calcifications affecting multiple lymph node chains is observed, two diseases are paramount for differential diagnoses: silicosis and sarcoidosis. Differentiation by imaging can be very difficult since both diseases can present with small nodules, conglomerate masses, and areas of emphysema, in addition to having a fibrosing evolution. In this situation, clinical and occupational history, such as previous contact with silica dust, is essential, since patients with silicosis, in general, carried out professional activities related to exposure. Our patient had no past or current history of exposure to silica, which allowed us to rule out the hypothesis of silicosis.

Sarcoidosis is a systemic inflammatory disease of unknown etiology, characterized by the formation of noncaseating epithelioid cell granulomas. Involvement of the lungs and mediastinal and hilar lymph nodes is more common, being observed in approximately 90% of patients, and is responsible for most of the morbidity and mortality associated with the disease. However, it can also involve any organ in the body; skin, eyes, heart, and liver are the most common ones. Pulmonary symptoms are variable. Patients may be asymptomatic and the disease may be identified on chest images obtained for unrelated reasons. However, patients more commonly present with nonspecific symptoms, such as cough, fatigue, and exertional dyspnea.^1^ ^,^ ^2^

On CT, the most typical findings of pulmonary involvement are small nodules with perilymphatic distribution, bilateral perihilar parenchymal opacities, and fibrotic changes. Hilar and/or mediastinal lymph node disease is also a common finding. Atypical manifestations, such as alveolar or mass-like opacities, honeycombing, miliary opacities, mosaic attenuation, and tracheobronchial involvement, may also be observed. Pleural effusion is rare in sarcoidosis.^1^ ^,^ ^2^ The final diagnosis was sarcoidosis.
